# Improving the measurement of maternal mortality in Thailand using multiple data sources

**DOI:** 10.1186/s12963-016-0087-z

**Published:** 2016-05-04

**Authors:** Worawan Chandoevwit, Phasith Phatchana, Kanjana Sirigomon, Kunakorn Ieawsuwan, Jutatip Thungthong, Saray Ruangdej

**Affiliations:** Faculty of Economics, Khon Kaen University, 123 Mitraphab Road, Muang, Khon Kaen 40002 Thailand; Thailand Development Research Institute, 565 Ramkhamhaeng Rd. Soi 39, Bangkok, Wangthonglang 10310 Thailand; National Health Security Office, The government complex commemorating His Majesty the King’s 80th birthday Anniversary 5th December, B.E. 2550 Building B, 120 Moo 3, Chaengwattana Road, Bangkok, Lak Si District 10210 Thailand

**Keywords:** Maternal mortality ratio, Maternal death, Pregnancy, Civil registration, Thailand

## Abstract

**Background:**

Thailand uses cause of death records in civil registration to summarize maternal mortality statistics. A report by the Department of Health using the Reproductive Age Mortality Studies (RAMOS) reported that the maternal mortality ratio (MMR) in 1997 was approximately three to four times higher than MMR based on the civil registration cause of death records. Here, we used multiple data sources to systematically measure maternal mortality in Thailand and showed a disparity between age groups and regions.

**Methods:**

We calculated the number of maternal deaths using a two-stage method. In the first stage, we counted the number of deceased mothers who successfully gave live births. In the second stage, we counted the number of women who died during the pregnancy, delivery, or the postpartum period without a live birth.

**Results:**

The number of maternal deaths dropped from 268 in 2007 to 226 in 2014. Nearly 50 % of the deaths occurred in Stage 1. The maternal mortality ratio in 2007 was 33.6 per 100,000 live births; the rate fell to 31.8 in 2014. The age ranges of women observed were 15-19, 20-24, 25-29, 30-34, 35-39, 40-44, and 45-49, and the MMR averages were 21.5, 23.8, 27.0, 42.1, 67.7, 115.4, and 423.4 per 100,000 live births, respectively. The Southern region consistently exhibited the highest MMR compared to other regions for every year analyzed, except 2012. Women in Bangkok had a lower risk of dying during pregnancy, delivery, and the postpartum period than women from other regions.

**Conclusions:**

We demonstrated that using multiple administrative data sources in the two-stage method was an efficient method that provided systematic measurement and timely reporting on the maternal mortality ratio. An additional benefit of the method was that information provided from the combined data sources, (e.g., the number of maternal deaths by age group and region) was relevant to the safe motherhood policy.

## Background

Thailand maintains two maternal mortality statistics from two government agencies in the Ministry of Public Health. First, the Bureau of Health Promotion (BHP) reported in 1990 that maternal mortality was 36.0 per 100,000 live births. This ratio declined until 1997, when it reached 14.2; it subsequently increased until it reached 28.0 by 2000 [1]. Second, the Bureau of Policy and Planning (BPP), which was later named the Bureau of Policy and Strategy (BPS), reported that the maternal mortality ratio in 1990 was 25.0 per 100,000 live births. They observed a decline to 7.0 in 1998 and a subsequent increase to 13.2 in 2000 [1]. The BPP reported values are nearly half of the BHP values.

UNICEF, in collaboration with BHP, conducted special studies in 1996 and 1997 [1, 2]. Using the Reproductive Age Mortality Studies (RAMOS) method [3], they found that the MMR was 44.3 in 1996 and it decreased to 36.5 in 1997 [2]. This report was approximately three to four times higher than the BPP report during the same time period [2]. The MMR was highest in the Southern region (65.1 per 100,000 live births) and lowest in the Central region (24.3 per 100,000 live births) [1]. Although the World Health Organization (WHO) and BHP recognize that RAMOS is a useful method for determining the MMR, it is time-consuming, complicated, and expensive to undertake on a large scale [1, 4].

A novel, inexpensive, and efficient method for measuring maternal mortality in Thailand was introduced by Chandoevwit et al. [5]. Using multiple data sources, the method showed that the MMR was 42 per 100,000 live births in 2006. In the same year, statistics from the BPS report were four times lower (12 per 100,000 live births) [6]. The pregnancy-related causes of death counted in Chandoevwit et al. are gathered from the civil registration and inpatient databases, but BPS used the causes of death from the civil registration alone. The cause of death in Thai civil registration was, however, criticized for its completeness and accuracy [7–9].

Existing methods of estimating MMR still leave considerable room for improvement. First, the maternal mortality statistics using the cause of death from civil registration could be inaccurate [7] or incomplete [8, 9]. Second, estimates obtained from statistical models provide MMR trends [4, 10], but they lack detailed information such as disparities of maternal death within domestic regions or across age groups. As a result, it is difficult to recommend effective safe motherhood strategies.

The objective of this study is to measure maternal mortality in Thailand using matched data from the civil registration database and inpatient diagnosis records. With rich information from multiple data sources, the major contribution of this study is a systematic measurement of maternal mortality that provides additional information on differences between age groups and regions in Thailand from 2007-2014.

## Method

### Definition

We adhered to the definition of maternal death from the International Statistical Classification of Diseases and Related Health Problems, Tenth Revision [11]. The WHO defines maternal death as “*The death of a woman while pregnant or within 42 days of termination of pregnancy, irrespective of the duration and site of the pregnancy, from any cause related to or aggravated by the pregnancy or its management but not from accidental or incidental causes*” [11]. Maternal deaths from either direct or indirect obstetric causes were included.

### Data sources

We used multiple data sources. First, civil registration data which includes birth and death information was retrieved. Second inpatient databases from the Central Office for Healthcare Information and the National Health Security Office (NHSO) were obtained. These two institutions administer inpatient data of two public health insurance schemes covering 83 % of the Thai population [12]. As of 2015, however, the NHSO is responsible for pooling national inpatient data of all public health insurance schemes. Data from multiple sources can be combined using the 13-digit Personal Identification Number (PID) that is assigned to every Thai citizen at birth.

### Measurement of the number of maternal deaths

**Fig. 1:**
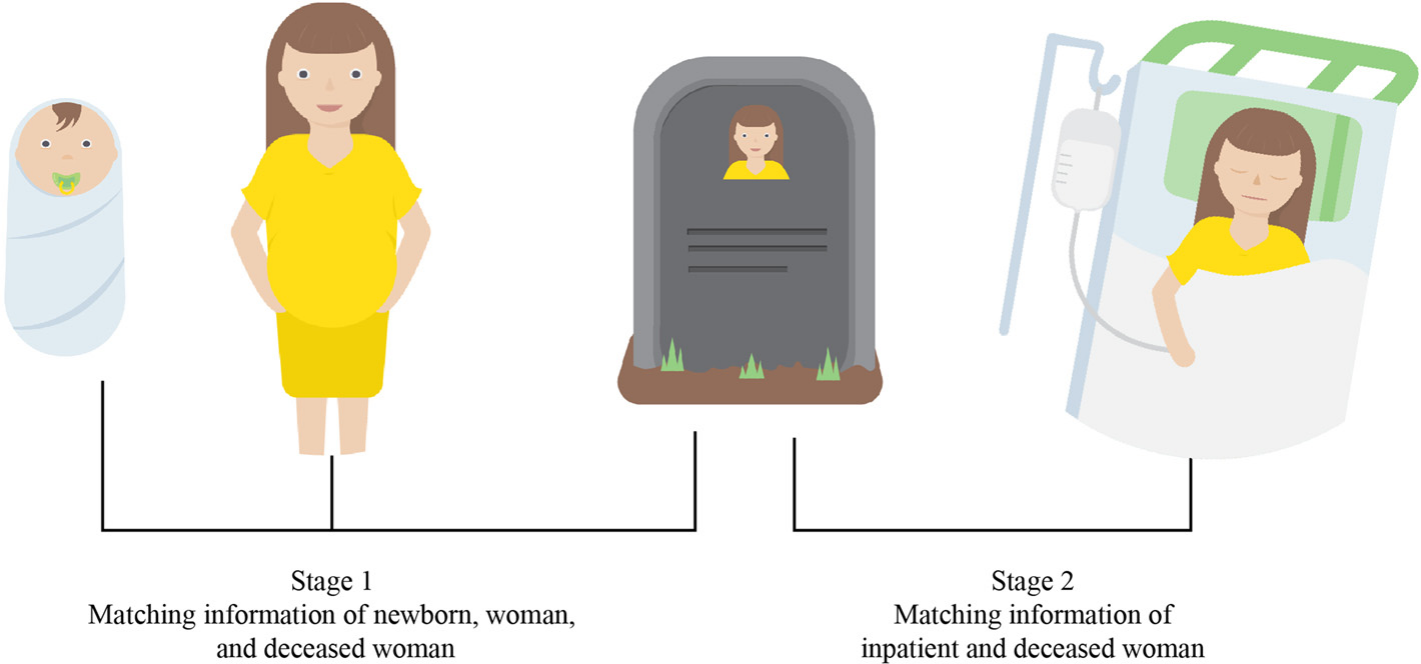
Linkage of multiple data sources

We calculated the number of maternal deaths using a two-stage method. In the first stage, we counted the number of deceased mothers who successfully gave live births. In the second stage, we counted the number of women who died during pregnancy, delivery, or the postpartum period without a live birth. Figure 1 depicts the information gathered for each subject from multiple data sources in Stage 1 and Stage 2.

#### Stage 1 mothers who died after giving a live birth

Data on the newborn and reproductive-aged deceased women from 2007-2014 were obtained from the civil registration database. Each newborn record contained a PID, the maternal PID, sex, date of birth, and place of birth. Additionally, the record of each deceased woman provided a PID, date of death, place of death, and the cause of death. The maternal PID of each newborn was matched with the deceased women PID. Matched records representing reproductive-aged women who gave live births were selected. Following the WHO [11] definition, we further screened for matched records in which the mother died within 42 days after the date of birth of the child. The inclusion and exclusion cases for this stage are given in Table 1.

**Table 1:** Data sources summary

	Stage 1: mothers who died after giving a live birth	Stage 2: pregnant women who died without a live birth
Data sources	- Civil registration containing data on birth and death information of the child and mother, respectively.	- Inpatient record from two public insurance schemes: CS and UCS^a^
	- Death certificate.	- Death certificate.
Result from matching multiple data sources	- Mothers who died within 42 days after the date of birth of their newborns.	- Women who were pregnant within a period of 270 days before their date of death.
		- Women who died within 42 days after child delivery or abortion.
Deceased women who are included in each stage	- All Thai citizens who gave live births at private or public hospitals or at home and register their newborns.	- Pregnant women who were hospitalized and used CS or UCS.
	- Ethnic minority who had a PID and gave live births.	
Deceased women who are excluded in each stage	- Unreported death	- Unreported death
	- Migrant workers who gave birth in Thailand.	- Pregnant women who had no hospital admission or did not use CS or UCS.
	- Ethnic minorities who did not have a PID.	- Pregnant women who gave birth at private hospitals. However, they should be included in stage 1 if they gave live birth.
	- Thai citizens who gave birth at home and did not register their newborns.	- Migrant workers and ethnic minorities.

^a^Thailand has three public health insurance schemes: *CS* for civil servants, their parents, and young children, *SS* for private employees and *UCS* for the rest of population

#### Stage 2 pregnant women who died without a live birth

The PIDs of reproductive-aged deceased women from the civil registration system were matched with the PIDs from the inpatient database. Each record of the inpatient database contained sex, age, date admitted, discharge date, primary and secondary diagnoses using ICD-10 codes, hospital code, and discharge code. Subjects with inpatient diagnosis codes containing O00-O99 (pregnancy, childbirth and the puerperium) within 270 days of the date of death were selected. Patients who died within 42 days after child delivery or abortive outcome were also selected for further screening. The selected records were reproductive-age deceased women who were pregnant within a period of 270 days before their date of death or who died within 42 days after child delivery or abortion. Some of the patients in the selected records had given live birth. These cases were matched with those from Stage 1 to identify overlapping records. The maternal diagnostic information was kept for further screening. The inclusion and exclusion cases from this stage are also given in Table 1.

In both stages, maternal deaths with accidental and incidental causes were not included. Excluding a subject based on either one or both conditions. First, the subjects causes of death identified in the death certificate must have been related to accidents, murder, intentional self-inflicted injury or poisoning and genocide. Second, the last inpatient records must have contained the following codes: S00-S99, T00-T79, T90-T98 (injury and poisoning of external causes), V01-X59 (accident), X60-X84 (intentional self-harm), X85-Y09 (assault), or Y10-Y36 (event of undetermined intent and legal intervention and operation of war).

## Results

Table 2 shows the number of maternal deaths from 2007-2014 using the two-stage method. Overall, in the eight-year period, the number of maternal deaths dropped 16 % from 268 in 2007 to 226 in 2014. The number of deaths from Stage 1 was approximately 50 % of the total. The MMR in 2007 was 33.6 per 100,000 live births, and it fell to 31.8 in 2014. From the 2007 to 2014 time period, MMR increased by 0.2 % annually. However, the official MMR increased from 12.2 per 100,000 live births in 2007 to 23.3 in 2014 with an average growth rate of 14.1 % annually.

**Table 2:** Number of maternal deaths in 2007-2014

Year	No. of maternal deaths			No. of LBs^a^	Maternal mortality per 100,000 LBs	Official maternal mortality per 100,000 LBs^a^
	Stage 1	Stage 2	Total			
2007	113	155	268	797,588	33.6	12.2
2008	177	156	333	784,256	42.5	11.3
2009	145	160	305	765,047	39.9	10.8
2010	152	146	298	761,689	39.1	10.2
2011	115	133	248	795,031	31.2	8.9
2012	120	126	246	801,737	30.7	17.6
2013	146	114	260	748,081	34.8	22.2
2014	130	96	226	711,805	31.8	23.3

^a^ *LBs* is live births. Data from NESDB [6, 23]

Table 3 gives the distribution of maternal mortality by age group. In the eight-year period, approximately 9.5 % of maternal deaths occurred in the 15-19 year-old group, 8.2 % occurred in the 40-44 year-old group, and 1.9 % of deaths occurred in the 45-49 year-old group. Older women had the highest risk of dying during pregnancy, delivery, and the postpartum period, as the MMRs of women in the 45-49 age group were much higher than women in the other age groups. The average MMR of women in each respective age group was: 20-24 years old: 23.8; 25-29 years old: 27.0; 30-34 years old: 42.1; and 35-39 years old: 67.7 per 100,000 live births. The MMR of women age 15-19 were lower than other age groups, except in the 2009-2011 period. The MMR by age group in 2009-2011 was a “J” shaped curve, which reflected results from 38 other countries, as shown in Blanc et al. [13].

**Table 3:** Maternal mortality ratio by age group

	Age group							
	15-19	20-24	25-29	30-34	35-39	40-44	45-49	Others^a^
2007								
MD^b^	19	40	62	66	55	21	5	
LBs^c^	116,086	196,390	215,888	163,888	80,129	19,043	1,320	4,844
MMR	16.4	20.4	28.7	40.3	68.6	110.3	378.8	
2008								
MD	24	59	72	76	69	31	2	
LBs	118,921	189,741	209,960	161,205	78,687	19,741	1,267	4,734
MMR	20.2	31.1	34.3	47.1	87.7	157.0	157.9	
2009								
MD	37	44	56	77	59	24	8	
LBs	119,828	184,096	203,387	156,397	76,340	19,036	1,266	4,697
MMR	30.9	23.9	27.5	49.2	77.3	126.1	631.9	
2010								
MD	32	48	61	74	52	25	6	
LBs	120,115	180,904	201,051	158,349	77,125	18,982	1,222	3,941
MMR	26.6	26.5	30.3	46.7	67.4	131.7	491.0	
2011								
MD	29	33	41	69	48	22	6	
LBs	129,321	186,942	204,684	167,671	80,348	20,089	1,293	4,683
MMR	22.4	17.7	20.0	41.2	59.7	109.5	464.0	
2012								
MD	20	44	51	66	43	19	3	
LBs	129,451	190,403	202,861	170,407	82,927	19,967	1,196	4,525
MMR	15.4	23.1	25.1	38.7	51.9	95.2	250.8	
2013								
MD	28	42	51	61	49	19	10	
LBs	121,960	177,873	183,315	160,404	79,923	19,227	1,190	4,189
MMR	23.0	23.6	27.8	38.0	61.3	98.8	840.3	
2014								
MD	19	40	38	55	54	18	2	
LBs	112,277	167,723	172,886	155,602	79,380	18,970	1,158	3,809
MMR	16.9	23.8	22.0	35.3	68.0	94.9	172.7	
Mean (95 % CI)	21.5 (18.0-25.0)	23.8 (21.2-26.3)	27.0 (24.0-29.9)	42.1 (38.8-45.3)	67.7 (60.6-74.9)	115.4 (101.4-129.5)	423.4 (270.7-576.2)	

^a^Others include age < 15, age > 49 and the unknown ages

^b^MD is maternal deaths

^c^Data from NESDB [24]

Table 4 shows a disparity in MMR across regions in Thailand. The total number of maternal deaths was highest in the Northeast, where the number of live births was also highest. MMRs in the Northeast fluctuated between 30.2 and 44.2 per 100,000 live births. The MMR in 2014 was about the same as the ratio in 2007. In 2012, the MMR in the Northeast increased 38.4 % in one year. For every year except 2012, the Southern region had the highest MMR compared to the other regions. The MMR in the Southern region showed a declining trend from 2008 to 2012. The average MMR in the Southern region was 47.6 (95 % CI, 43.5-51.7) per 100,000 live births. The maternal mortality in the Northern region improved from 2009 to 2012, as the MMR declined from 38.7 to 22.4 per 100,000 live births. However, the MMR increased in 2013 and 2014, almost returning to 2007 ratios. Women in Bangkok have had a lower risk of dying during pregnancy, delivery, and the postpartum period than women from other regions. The average MMR for Bangkok was 20.2 (95 % CI, 15.4-25.1) per 100,000 live births.

**Table 4:** Maternal mortality ratio by region

	Region				
	North	Northeast	Central	South	Bangkok
2007					
MD	34	75	75	69	15
LBs^a^	114,705	223,604	211,234	137,823	110,222
MMR	29.6	33.5	35.5	50.1	13.6
2008					
MD	37	97	94	80	25
LBs	111,558	219,434	209,044	137,565	106,655
MMR	33.2	44.2	45.0	58.2	23.4
2009					
MD	43	75	84	70	33
LBs	111,057	216,893	201,604	134,381	101,112
MMR	38.7	34.6	41.7	52.1	32.6
2010					
MD	41	90	77	65	25
LBs	114,501	215,605	199,877	133,563	98,143
MMR	35.8	41.7	38.5	48.7	25.5
2011					
MD	34	69	62	58	25
LBs	114,146	228,195	210,293	141,378	101,019
MMR	29.8	30.2	29.5	41.0	24.7
2012					
MD	26	95	56	56	13
LBs	116,014	227,213	211,742	143,488	103,280
MMR	22.4	41.8	26.4	39.0	12.6
2013					
MD	26	77	72	68	17
LBs	108,048	213,184	194,471	138,549	93,829
MMR	24.1	36.1	37.0	49.1	18.1
2014					
MD	30	68	63	55	10
LBs	103,909	203,661	187,758	129,235	88,242
MMR	28.9	33.4	33.6	42.6	11.3
Mean (95 % CI)	30.3 (26.7-33.9)	37.0 (33.7-40.2)	35.9 (31.9-39.8)	47.6 (43.5-51.7)	20.2 (15.4-25.1)

^a^Data from NESDB [23]

## Discussion

This study combined data from civil registration and inpatient diagnoses to identify the number of maternal deaths in Thailand in 2007-2014 and calculate the MMR. We found that the maternal mortality ratios calculated using the two-stage method were about three to four times higher than the official MMR reported by the BPS in 2007-2011 [6]. The size of the difference was similar to the results presented in Kanshana et al. [1]. The gaps between our calculation and the BPS’s report diminished in 2012-2014. The official report showed a shift of MMR during this period. Moreover, this study showed that the MMR in Thailand was lower than that presented by Kassebaum et al. [14]. Using statistical modeling techniques, they estimated that the MMR in Thailand increased from 43.6 per 100,000 live births in 1990 to 89.6 in 2003 and dropped to 69.5 in 2013.

Using rich data from multiple sources, we were able to demonstrate the variation of MMR by age group and region. In Thailand, the risk of death during pregnancy or childbirth increased with age. This pattern was similar to that observed in other Southeast Asian countries [15]. The oldest age cohorts were exposed to the highest risk of death during pregnancy, childbirth, and puerperium, while adolescent women were exposed to the lowest risk. The distribution of maternal deaths was most highly concentrated in the 30-34 and 35- 39 year-old groups. Focusing policy attention on these age groups could effectively reduce the MMR.

The variations in maternal death by regions shown in this study were similar to results presented by Kanshana et al. [1]. The Southern region of Thailand had the highest MMR compared to other regions. Possible reasons for this disparity were cultural differences and unequal access to health services, which might lower skilled birth attendance among pregnant women in the South. However, the gaps between regions seemed to be diminishing. The MMR in the Southern region reduced by 1.3 % annually from 2007-2014, while the national ratio increased by 0.2 %. In 1997, the MMR in the Southern region was 78.4 % higher than the national MMR [1]. In 2007, it was 49.0 % higher than the national MMR, but this rate differential reduced to 34.0 % in 2014. Although the Thai MMR declined at a slow rate from 1997 [1] to 2014, the disparity between regions improved.

One limitation of this study was that the deaths occurring during pregnancy, childbirth, and puerperium could be underreported, as shown in Table 1. Two types of underreporting might have happened. The first was due to underregistration of deaths and the second was due to the misclassification of the cause of death. Vapattanawong and Prasertkul estimated that the percentage of unregistered deaths of Thai females age 15-59 was 14.8 % [16]. The number of unregistered deaths among female migrant worker was unknown. Given the available evidence, this figure was presumed to be an upper limit of an underestimating of maternal deaths due to underregistration of deaths among Thai women because it included all causes of death and also included females age 50-59 who were above the reproductive age.

In this study, we used inpatient diagnostic data to rectify the misclassification of maternal deaths in stage 2. The causes of death certified in the death registration in Thailand were incomplete or of poor quality [7, 9, 17–19]. About 35-40 % of registered deaths were ill-defined [17]. Moreover, of those who died in hospitals, about 51 % of sampling audited death certificates contained certification errors [18].

Inpatient diagnostic data used in the present study were obtained from two public health insurance schemes covering civil servants and all non-private employees. Therefore, the second stage of our method did not include deceased women who used to work as private employees; the samples did not include women who were under the public health insurance coverage by the Social Security Office (SSO). In 2007-2014, 27 % of reproductive-aged women were covered by this health insurance scheme. Including all causes of deaths, the proportion of SSO reproductive-aged females to the total reproductive-aged females was approximately 16 % [20, 21]. This could be used as a rough figure for adjusting the number of the maternal death misclassified in stage 2. At present, we do not have enough information to estimate the proportion of maternal deaths under the SSO health insurance scheme. With an improved pooling of the national inpatient databases in the future, this shortcoming could be remedied.

Another reason for potential underreporting due to misclassification of the cause of death was childbirths occurred outside public or private hospitals. This accounted for 6 % on average from 2007-2014 [21, 22]. If these women died during childbirth or the puerperium, the causes of death on their death certificates could have been ill-defined [7, 8, 19] or unrelated to pregnancy. Using the data from the present study, we found that only 21.7 % of all maternal deaths had pregnancy-related causes of death in their death certificates. An improvement of the quality of death registration could reduce the misclassification errors.

Despite these limitations, the two-stage method using multiple data sources can provide additional useful information than was beyond the scope of this study. For example, a future study could use information retrieved from the inpatient database to analyze the cause of death and answer other important questions.

## Conclusion

We demonstrated that using multiple administrative data sources in the two-stage method was an efficient method that provided systematic measurement and timely reporting on the maternal mortality ratio. An additional benefit of the method was that information provided from the combined data sources, (e.g., the number of maternal deaths by age group and region) was relevant to the safe motherhood policy.

## Abbreviations

BHP: Bureau of Health Promotion
BPS: Bureau of Policy and Strategy
CHI: Central Office for Healthcare Information
CI: confidence interval
CS: civil servant medical benefit scheme
ICD-10: International Statistical Classification of Diseases and Related Health Problems (10^th^ edition)
LBs: live births
MD: maternal deaths
MMR: maternal mortality ratio
NESDB: National Economic and Social Development Board
NHSO: National Health Security Office
PID: Personal Identification Number
RAMOS: Reproductive Age Mortality Studies
SS: social security scheme
SSO: Social Security Office
UCS: universal coverage scheme

## References

[1] Kanshana S, Amornwichet P, Nuntamanop S, Kullerk N (2002). Maternal mortality in Thailand 1997-1998.

[2] Bureau of Health Promotion (2006). Confidential enquiries into maternal deaths in Thailand.

[3] AbouZahr C, Wardlaw T (2004). Maternal Mortality in 2000: Estimates developed by WHO, UNICEF and UNFPA.

[4] WHO/UNICEF/UNFPA/World Bank/the United Nations Population Division (2014). Trends in maternal mortality: 1990-2013.

[5] Chandoevwit W, Kasitipradith N, Soranastaporn S, Vacharanukulkieti K, Wibulpolprasert S: Using multiple data for calculating the maternal mortality ratio in Thailand. TDRI Quarterly Review 2007. http://tdri.or.th/wp-content/uploads/2012/09/t5s2007002.pdf. Accessed 1 Sept 2015.

[6] National Economic and Social Development Board: Maternal mortality per 100,000 live births in 2001-2014. http://social.nesdb.go.th/SocialStat/StatReport_Final.aspx?reportid=216&template=1R2C&yeartype=M&subcatid=15. Accessed 4 Feb 2016.

[7] Tangcharoensathien V, Faramnuayphol P, Teokul W, Bundhamcharoen K, Wibulpholprasert S (2006). A critical assessment of mortality statistics in Thailand: potential for improvements. Bull World Health Organ.

[8] Phillps D, Lozano R, Naghavi M, Atkinson C, Gonzalez-Medina D, Mikkelsen L, Murray CJ, Lopez AD (2014). A composite metric for assessing data on mortality and causes of death: the vital statistics performance index. Popul Health Metr.

[9] Rao C, Porapakkham Y, Pattaraarchachai J, Polprasert W, Swampunyalert N, Lopez AD (2010). Verifying causes of death in Thailand: rationale and methods for empirical investigation. Popul Health Metr.

[10] Lozano R, Wang M, Foreman KJ, Rajaratnam JK, Naghavi M, Marcus JR, Dwyer-Lindgren L, Lofgren KT, Phillps D, Atkinson C (2011). Progress towards Millennium Development Goals 4 and 5 on maternal and child mortality: an updated systematic analysis. Lancet.

[11] World Health Organization (2004). International Statistical Classification of Diseases and Related Health Problems: Tenth Revision.

[12] National Economic and Social Development Board: Coverage of public health insurance schemes in Thailand in 2002-2014. http://social.nesdb.go.th/SocialStat/StatReport_Final.aspx?reportid=165&template=2R1C&yeartype=M&subcatid=46. Accessed 19 Sept 2015.

[13] Blanc A, Winfrey W, Ross J. New findings for maternal mortality age patterns: aggregated results for 38 countries. PLoS One. 2013;8:e59864. 10.1371/journal.pone.0059864 PMC3629034 23613716

[14] Kassebaum NJ, Bertozzi-Villa A, Coggeshall MS, Shackelford KA, Steiner C, Heuton KR, Gonzalez-Medina D, Barber R, Huynh C (2014). Global, regional, and national levels and causes of maternal mortality during 1990-2013: a systematic analysis for the Global Burden of Disease Study 2013. Lancet.

[15] Nove A, Matthews Z, Camacho AV (2014). Maternal mortality in adolescents compared with women of other ages: evidence from 144 countries. Lancet Global Health.

[16] Vapattanawong P, Prasartkul P. Under-registration of deaths in Thailand in 2005-2006: results of cross-matching data from two sources. Bull World Health Organ. 2011;89:806-812. 10.2471/BLT.10.083931 PMC3209718 22084526

[17] Porapakkham Y, Rao C, Pattaraarchachai J, Polprasert W, Vos T, Adair T, Lopez AD (2010). Estimated causes of death in Thailand, 2005: implications for health policy. Popul Health Metr.

[18] Pattaraarchachai J, Rao C, Polprasert W, Porapakkham Y, Pao-In W, Singwerathum N, Lopez AD (2010). Cause-specific mortality patterns among hospital deaths in Thailand: validating routine death certification. Popul Health Metr.

[19] Polprasert W, Rao C, Adair T, Pattaraarchachai J, Porapakkham Y, Lopez AD (2010). Cause-of-death ascertainment for deaths that occur outside hospitals in Thailand: application of verbal autopsy methods. Popul Health Metr.

[20] Social Security Office (2015). Social security statistics 2014.

[21] Bureau of Policy and Strategy (2015). Public health statistics 2014.

[22] Bureau of Planning and Strategy (2011). Public Health Statistics 2011.

[23] National Economic and Social Development Board: Number of live births by region in 2001-2014. http://social.nesdb.go.th/SocialStat/StatReport_Final.aspx?reportid=213&template=2R2C&yeartype=M&subcatid=15. Accessed 4 Feb 2016.

[24] National Economic and Social Development Board: Fertility rate by age group in 2002-2014. http://social.nesdb.go.th/SocialStat/StatReport_Final.aspx?reportid=215&template=1R2C&yeartype=M&subcatid=15. Accessed 4 Feb 2016.

